# Technical and anatomical challenges to approach robotic-assisted radical prostatectomy in patients with Urolift^®^

**DOI:** 10.1590/S1677-5538.IBJU.2023.9905

**Published:** 2023-02-15

**Authors:** Marcio Covas Moschovas, David Grant Loy, Abdel Jaber, Shady Saikali, Travis Rogers, Sarah Kind, Vipul Patel

**Affiliations:** 1 AdventHealth Global Robotics Institute FL USA AdventHealth Global Robotics Institute, FL, USA

## Abstract

**Introduction:**

Urolift® is a surgical modality to treat lower urinary tract symptoms (LUTS) in patients with enlarged prostates (1). However, the inflammatory process caused by the device usually displaces the prostate’s anatomical landmarks and challenges surgeons performing robotic-assisted radical prostatectomy (RARP). In this video, we will illustrate several technical challenges in patients with Urolift ® who underwent RARP.

**Material and Methods:**

We performed a video compilation with several surgical steps illustrating key aspects and critical details of the anterior bladder neck access, lateral bladder dissection from the prostate, and posterior prostate dissection to avoid ureteral and neural bundles injuries.

**Results:**

We perform our RARP technique with our standard approach in all patients (2-6). The beginning of the case is performed like every patient with an enlarged prostate. We first identify the anterior bladder neck and then complete its dissection with Maryland and Scissors. However, extra care must be taken in the anterior and posterior bladder neck approach due to the clips found during the dissection. The challenge starts when opening the lateral sides of the bladder until the base of the prostate. It is crucial to perform the bladder neck dissection beginning at the internal plane of the bladder wall. Such dissection is the easiest way to recognize the anatomical landmarks and potential foreign materials, such as clips, placed during previous surgeries. We cautiously work around the clip to avoid using cautery on the top of the metal clips because energy is transmitted from one edge to the other of the Urolift ®. This can be dangerous if the edge of the clip is close to the ureteral orifices. The clips are usually removed to minimize cautery conduction energy. Finally, after isolating and removing the clips, the prostate dissection and subsequent surgical steps are continued with our conventional technique. Before proceeding, we ensure that all clips are removed from the bladder neck to avoid complications during the anastomosis.

**Conclusions:**

Robotic-assisted radical prostatectomy in patients with Urolift ® is challenging due to modified anatomical landmarks and intense inflammatory processes in the posterior bladder neck. When dissecting the clips placed next to the base of the prostate, it is crucial to avoid cautery because energy conduction to the other edge of the Urolift ® can cause thermal damage to the ureters and neural bundles.
